# Peptide Inhibitor of Complement C1, a Novel Suppressor of Classical Pathway Activation: Mechanistic Studies and Clinical Potential

**DOI:** 10.3389/fimmu.2014.00406

**Published:** 2014-08-22

**Authors:** Julia A. Sharp, Pamela H. Whitley, Kenji M. Cunnion, Neel K. Krishna

**Affiliations:** ^1^Department of Microbiology and Molecular Cell Biology, Eastern Virginia Medical School, Norfolk, VA, USA; ^2^American Red Cross, Mid-Atlantic Region, Norfolk, VA, USA; ^3^Department of Pediatrics, Eastern Virginia Medical School, Norfolk, VA, USA; ^4^Children’s Specialty Group, Division of Infectious Diseases, Norfolk, VA, USA

**Keywords:** complement, classical pathway, C1q, MBL, peptide, inhibitor, ABO incompatibility, AIHTR

## Abstract

The classical pathway of complement plays multiple physiological roles including modulating immunological effectors initiated by adaptive immune responses and an essential homeostatic role in the clearance of damaged self-antigens. However, dysregulated classical pathway activation is associated with antibody-initiated, inflammatory diseases processes like cold agglutinin disease, acute intravascular hemolytic transfusion reaction (AIHTR), and acute/hyperacute transplantation rejection. To date, only one putative classical pathway inhibitor, C1 esterase inhibitor (C1-INH), is currently commercially available and its only approved indication is for replacement treatment in hereditary angioedema, which is predominantly a kinin pathway disease. Given the variety of disease conditions in which the classical pathway is implicated, development of therapeutics that specifically inhibits complement initiation represents a major unmet medical need. Our laboratory has identified a peptide that specifically inhibits the classical and lectin pathways of complement. *In vitro* studies have demonstrated that these peptide inhibitors of complement C1 (PIC1) bind to the collagen-like region of the initiator molecule of the classical pathway, C1q. PIC1 binding to C1q blocks activation of the associated serine proteases (C1s–C1r–C1r–C1s) and subsequent downstream complement activation. Rational design optimization of PIC1 has resulted in the generation of a highly potent derivative of 15 amino acids. PIC1 inhibits classical pathway mediated complement activation in ABO incompatibility *in vitro* and inhibiting classical pathway activation *in vivo* in rats. This review will focus on the pre-clinical development of PIC1 and discuss its potential as a therapeutic in antibody-mediated classical pathway disease, specifically AIHTR.

## Introduction

Activation of the classical pathway of complement is mediated via C1, a multimolecular complex composed of the recognition molecule C1q and the associated serine proteases C1r and C1s (1). IgM or clustered IgG serves as the principal ligand for C1q, resulting in the sequential activation of C4 and C2 to form the classical pathway C3-convertase leading to C3 and C5 activation. This cascading activation sequence generates a number of inflammatory effector molecules designed to limit infection by invading pathogens. Additionally, C1 plays an essential housekeeping role by recognizing and clearing cellular debris, immune complexes, altered self, and apoptotic cells (2) as well as identifying abnormal structures such as beta-amyloid fibrils (3, 4) and the pathological form of the prion protein (5, 6). The ability of this molecule to distinguish self from non-self is critical for immune tolerance and homeostasis (7).

**Figure 1:**
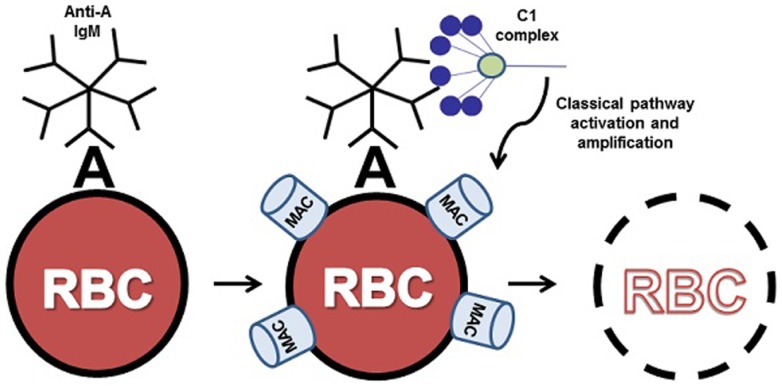
**ABO incompatible destruction of RBCs by the classical pathway of complement**. In this example, transfused cells expressing A antigen have been exposed to incompatible recipient serum containing anti-A IgM. The bound IgM molecule is then recognized by serum complement C1 complex. Upon binding to IgM, C1 activates the classical pathway, resulting in complement deposition on the donor cell and eventual clearance or lysis of host RBCs by the membrane attack complex (MAC). For clarity, only C1 binding and MAC formation are illustrated here.

Under normal circumstances, complement activation is tightly controlled by a number of regulatory proteins to minimize host tissue damage. However, aberrant activation of the classical, lectin, or alternative pathways of complement leads to significant host tissue damage in many inflammatory diseases (8). In the case of the classical pathway of complement, irregular activation of this pathway has been implicated in cold agglutinin disease (CAD) (9), acute/hyperacute transplantation rejection (10), and other inflammatory and autoimmune conditions (11). In the field of hematology, inappropriate activation or dysregulated control of complement activation plays a central role in hemolytic disease processes such as acute intravascular hemolytic transfusion reaction (AIHTR) (12), autoimmune hemolytic anemia (13), and hemolytic disease of the newborn (14). In these disease processes, preexisting antibodies, typically natural antibodies, bind to erythrocyte surface antigens initiating classical complement pathway activation via C1 (15). Subsequent terminal complement cascade activation leads to membrane attack complex (MAC) formation on the erythrocyte surface and intravascular hemolysis. In the case of an AIHTR (such as an ABO incompatible transfusion), recipient serum containing antibody that specifically recognizes antigens on the surface of the donor’s red blood cells (RBCs) results in classical pathway mediated rapid intravascular hemolysis of donor RBCs within minutes (Figure 1). Due to the rapidly amplifying cascade driving this process, AIHTR frequently results in shock, disseminated intravascular coagulation, acute renal failure due to hemoglobinuria, and death (16). To date, there are no pharmacological interventions to treat the underlying mechanisms of this disease processes such that treatment is primarily supportive (17, 18). Thus, there is a critical need for inhibitors of the classical pathway that can block complement activation at the level of C1 and prevent the avalanche of complement activation at the initiation event of the cascade. In this review, we describe the identification and characterization of a novel inhibitor of C1 termed peptide inhibitor of complement C1 (PIC1) and discuss its potential as an anti-complement therapeutic for transfusion medicine applications.

## Discovery and Characterization of PIC1

The findings that led to the discovery of the complement inhibitory peptide PIC1 in our laboratory initially came from our research program on human astrovirus capsid assembly. Below, we discuss how PIC1 has evolved from a capsid protein of 787 amino acids to a 15 amino acid peptide that is unique in nature.

### Human astrovirus coat protein inhibition of the classical and lectin pathways

Human astroviruses (HAstVs) are a major cause of diarrhea in human infants (19). The virus replicates in intestinal epithelial cells, but produces no inflammation in the intestinal tissues (20). We showed in hemolytic complement assays that lysates made from cell culture infected with HAstV serotypes 1, 2, 3, and 4 all strongly inhibited serum complement activation, >84% (21). Human astrovirus type 1 (HAstV-1) is the most common serotype worldwide (22). Recombinantly expressed HAstV-1 coat protein (CP) was able to inhibit complement-mediated hemolysis >90%. Subsequent testing showed that CP was able to inhibit the activation of C3, C5, and C5b-9 in serum, demonstrating that the generation of downstream effectors was inhibited (21). CP strongly inhibited activation of the classical pathway and antibody-initiated complement activation in a variety of systems, but had no significant effects on the alternative pathway. CP inhibited activation of C1, as assayed by C1s activation, preventing the activation of C4 in serum. Moreover, complement activation could be restored with the addition of exogenous C1. CP bound to both the globular head regions and collagen-like regions (CLRs) of C1q, but had higher affinity for the CLR, where the C1s–C1r–C1r–C1s tetramer is nestled. C1s was displaced from intact C1 by CP, suggesting the displacement of the cognate serine protease(s) is the likely mechanism of inhibiting C1 activation (23). CP also inhibited mannan activation of MBL/MASP2 in human serum (23). MBL with a point mutation that prevents binding to MASP2 (i.e., Lys55Gln substitution) cannot bind CP, suggesting that CP competes for the cognate serine protease binding site of MBL, similar to C1 (23).

### PIC1 inhibition of antibody-initiated complement activation

**Figure 2:**
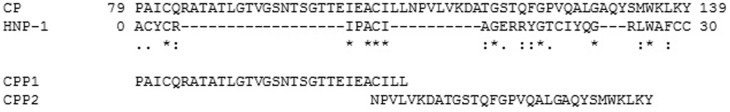
**Alignment of CP with the HNP-1 molecule using ClustalW analysis**. Homologous residues are identified as follows: identical (*), conserved (:), and semi-conserved (.) residues. CPP1 and CPP2 were synthesized based upon this alignment.

Within the amino acid sequence of the CP molecule, we identified a 60-residue region of homology corresponding to human neutrophil peptide 1 (HNP-1) (Figure 2). HNP-1 has been identified by other investigators as an inhibitor of C1 and MBL (24, 25). From the region of homology found within the CP molecule, two synthetic peptides were generated of 30 residues each (Figure 2). The PIC1 derivative coat protein peptide 1 (CPP1) inhibited the binding of CP to C1q and inhibited C1 activation, as assayed by C1s cleavage, suggesting a similar mechanism of action as the parent compound (Table 1) (26). Coat protein peptide 2 did not demonstrate either of these properties, indicating that the active region of CP could be successfully narrowed to a small peptide construct. A further derivative of CPP1 with an alanine substitution at position 23 (E 23A), as indicated by bold red in Table 1, demonstrated superior inhibitory activity to CPP1 (26). Additional truncations demonstrated that removing the central region of CPP1 (Δ8–22) yielded a functional peptide of 15 residues (Table 1) (26). The Δ8–22 construct inhibited antibody-initiated complement activation to a greater degree than CPP1, as demonstrated in hemolytic as well as C4 activation assays and similar to the parent CP. As a smaller peptide with similar potency to the E23A construct, Δ8–22 still had minimal effect on the alternative pathway (26).

**Table 1:** **PIC1 derivatives**.

Peptide	Peptide sequence (N → C)	Classical pathway inhibition values using 0.77 mM peptide (%)
CPP1	PAICQRATATLGTVGSNTSGTTEIEACILL	50
E23A	PAICQRATATLGTVGSNTSGTT 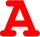 IEACILL	80
Δ8–22	PAICQRAEIEACILL	70
PA	IALILEPICCQERAA	85

### Further optimization of PIC1 derivatives

**Figure 3:**
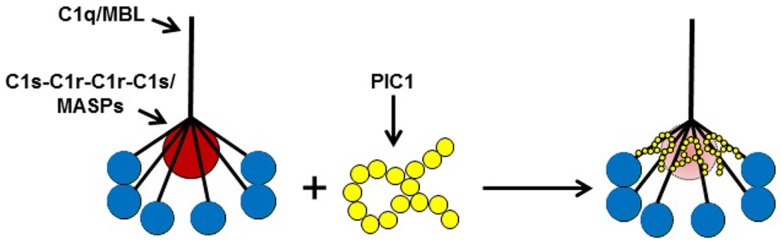
**A model of PIC1 interfering with the normal association of C1q/MBL with their cognate serine proteases (C1s–C1r–C1r–C1s/MASPs)**. Our data suggest PIC1 functionally disrupts the C1s–C1r–C1r– C1s/MASPs interaction with collagen-like region (CLR) of C1q/MBL, respectively. By virtue of PIC1 binding to this region of the CLR, the serine proteases are hypothesized to be in suboptimal conformation for autoactivation and initiation of the classical and/or lectin pathways of complement.

In an attempt to further optimize the PIC1 Δ8–22 derivative and improve its solubility properties, the peptide sequence was rearranged such that most of the hydrophobic residues were placed at the N terminus and the hydrophilic residues were placed at the C terminus. The rearranged PIC1 derivative was termed polar assortant (PA) (Table 1) and demonstrated superior complement inhibitory activity to all other derivatives previously reported (27). A deletion analysis of PA showed that loss of one amino acid residue from the N or C terminus destroyed inhibitory activity suggesting that 15 residues may be the optimal length of this PIC1 derivative (27). An alanine scan and a scan substituting each residue of PA with a negative or positively charged amino acid (40 total substitutions analyzed) revealed that PA was the most potent PIC1 analog capable of inhibiting classical pathway mediated complement activation [Ref. (27), and data not shown]. PA was also demonstrated to specifically bind to the CLR of C1q by ELISA and bound to purified C1q with a mean equilibrium dissociation constant (K_D_) of 33.3 nM as determined by surface plasmon resonance (27). It has been previously demonstrated by surface plasmon resonance that immobilized C1q binds to its cognate serine proteases (C1s–C1r–C1r–C1s) with a K_D_ of 2.72 nM (28). The binding of PA to C1q with similar affinity for C1s–C1r–C1r–C1s (i.e., nM range) is consistent with our hypothesis that the PIC1 peptides may function by displacing, at least partially, C1s–C1r–C1r–C1s from the CLR of C1q thereby changing the optimal conformation of the serine protease tetramer and preventing autoactivation. A hypothetical model of the mechanism by which PIC1 derivatives inhibit C1 and MBL activation is presented in Figure 3.

**Figure 4:**
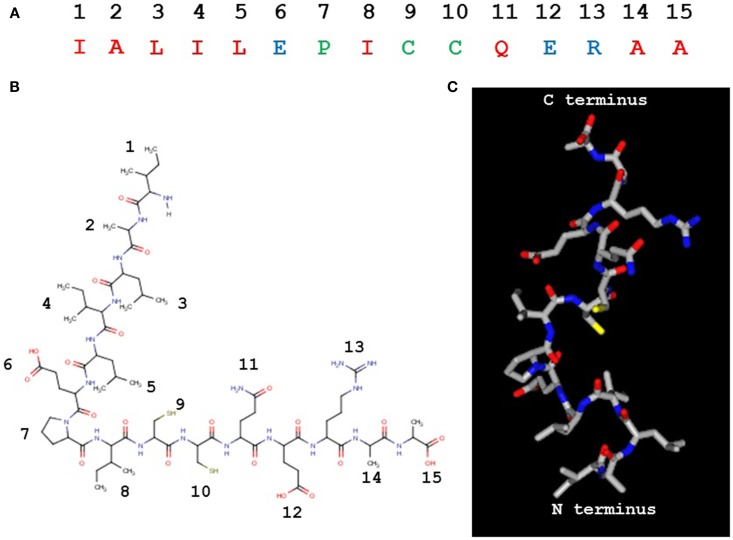
**Peptide inhibitors of complement C1 derivative PA**. **(A)** The 15 residue PA molecule is made up of 9 hydrophobic (red), 3 polar/charged (blue), and 3 intermediary (green) amino acid residues. **(B)** A linear version of the PA molecule illustrating the amino acid side chains. **(C)** A structural model of PA. **(B,C)** Created in MarvinView.

The PIC1 PA derivative, to our knowledge, is unique in nature (Figures 4A,B) as it does not possess any identifiable similarity with known proteins by NCBI blast search. It is 15 residues in length with a pI of 4.53 and a molecular weight of 1643 Da. While our data to date suggest that PA disrupts the interaction of C1s–C1r–C1r–C1s and MASP2 with the CLR of C1q and MBL, respectively, the precise molecular details of this interaction are unclear. As mentioned above, astrovirus CP does not bind an MBL mutant in which the lysine residue that are critical for MASP2 binding to the CLR of MBL has been substituted with another residue (23). This suggests that PIC1 derivatives may bind to the same critical lysine residue on the MBL and C1q molecules. While it is obvious to suggest that this reactive lysine residue may interact with either of the negatively charged glutamic acid residue on PA at positions 6 and 12 (Figures 4A,B), substitution of either of these residues individually or together with alanine had no detrimental effect on PA inhibitory activity (27), suggesting that binding of these derivatives to C1q was not compromised. It is highly conceivable that PIC1 may bind to the C1q/MBL CLR through other interactions such as hydrophobic and hydrogen bonding. Due to the large number of hydroxylated residues that exist in the CLR of C1q and MBL, extensive hydrogen bonding with PIC1 is an entirely feasible mechanism by which PIC1 may bind these molecules. Studies of PIC1 interaction with C1q using protein footprinting techniques combined with mass spectrometry are currently underway in our laboratory. The recent development of a system to produce the A, B, and C chains of C1q recombinantly (29) also provides a means to produce specific mutations in the C1q molecule, which would provide an invaluable resource in mapping the regions of interaction between C1q and PIC1. Precise details on the interaction of PIC1 with C1q and MBL would also benefit from structural studies utilizing X-ray crystallography and/or nuclear magnetic resonance imaging to directly visualize structural interactions. A hypothetical model of PIC1 PA is shown in Figure 4C.

## Pre-Clinical Development of PIC1

### PIC1 inhibition of ABO incompatibility *in vitro* and *in vivo* inhibition of complement

To evaluate the ability of PIC1 derivatives to inhibit ABO mediated RBC lysis, two PIC1 analogs were tested in an *in vitro* model of ABO incompatibility. E23A and an acetylated version of PA were both demonstrated to dose-dependently inhibit lysis of human AB RBCs incubated with human O serum in a modified hemolytic assay (27). Acetylated PA has identical inhibitory activity compared to unmodified PA (27). To preliminarily assess the *in vivo* complement suppression profile of these two derivatives, 20 mg of E23A and an acetylated version of PA were injected into 250 g male Wistar rats. Both peptides were able to cross the species barrier and inhibit serum complement activity in these animals as assessed by hemolytic assay using serum purified from the blood *ex vivo*. Administration of the acetylated PA molecule resulted in *in vivo* complement suppression up to 24 h post-injection (27). These findings demonstrate that PIC1 molecules have excellent potential for pre-clinical testing in small animal models of antibody-initiated complement disease.

### Development of a rat model of AIHTR

A simple yet elegant model of complement-mediated AIHTR in rodents has been previously reported (30, 31). In this mouse model, developed in the laboratory of Dr. K. Yazdanbakhsh (32–34), human RBCs fluorescently labeled with the dye PKH-26 were transfused i.v. via tail vein and complement-mediated hemolysis of the transfused cells analyzed. In this mouse strain, natural antibodies directed against antigens on the human RBCs initiated complement activation leading to rapid lysis over 120 min. Soluble complement receptor 1 (sCR1) or derivatives of this molecule were demonstrated to temporarily inhibit hemolysis as well as C3 and C4 deposition on the transfused human RBCs (32). In the past few years, sCR1 (also known as TP-10) has been explored in clinical trials of human diseases; however, these trials have been discontinued for various reasons. In order to test PIC1 in a pre-clinical model of AIHTR, we have recently developed a Wistar rat transfusion model (35). The rationale for utilizing the rat is threefold: (i) the larger size of the rat provides adequate blood volume to perform multiple blood draws for analysis post-transfusion, (ii) extracted mouse serum is notoriously difficult to use in *ex vivo* hemolytic assays (36), and (iii) while human RBCs are identical in size to rat RBCs, they are twice the size of mouse RBCs and thus may have difficulty in transiting the narrow capillaries of the mouse (37). Wistar rats are also thought to have natural antibodies against human erythrocytes (38).

To establish this model, we first determined *in vitro* that Wistar rat serum lysed human RBCs in an antibody-initiated, classical complement pathway dependent manner by utilizing complement sufficient or complement-deficient Wistar rat serum in the presence and absence of naturally occurring anti-human RBC antibody (35). This was achieved by coating human AB RBCs with complement-deficient Wistar rat serum that contains antibodies to the human RBCs. When the antibody coated RBCs were exposed to antibody deficient, complement sufficient Wistar rat serum, the RBCs were lysed in a dose-dependent manner (i.e., increasing the amount of antibody on the surface of the RBCs increased lysis by rat serum). Thus, lysis required both anti-human erythrocyte antibodies from the Wistar rat serum and activatable rat complement. Using various buffers, we demonstrated that the lysis of the RBC *in vitro* was due to classical pathway activation. To study the role of complement in acute intravascular hemolysis *in vivo*, Wistar rats were treated either with or without cobra venom factor (CVF) to deplete complement activity. PHK-26 labeled or unlabeled human AB RBCs were then injected into both groups of rats, followed by serial blood draws up to 2 h. Venous blood clearance and lysis of transfused RBCs at each time point was measured by flow cytometry (FITC labeled anti-human CD235a was used to detect unlabeled human cells) and spectrophotometry for free hemoglobin. Transfusion of human RBCs into rats showed significantly less hemolysis in the CVF group versus untreated group for both PHK-26 labeled and unlabeled cells by both flow cytometry and spectrophotometry (35). RBC sequestration was determined in the liver, spleen, and kidney by immunohistochemistry 2 h post-transfusion and demonstrated no quantitative difference for extravascular RBCs in the tissues between the two groups for the liver and spleen (35). However, intravascular human RBCs in the kidney glomeruli were different between the two groups, consistent with the flow cytometry and spectrophotometry data. Given these findings, we believe this simple rat model is ideal for testing novel inhibitors of classical pathway activation such as PIC1 for the prevention and treatment of AIHTR. Experiments to test PIC1 in this model are currently underway in our laboratory.

## Complement Inhibitors for Transfusion Medicine Applications

Acute hemolytic transfusion reactions occur in almost one-fifth of total transfusions, with life-threatening reactions in approximately 0.5% (39). The clinical presentation of acute hemolytic transfusion reactions is broad, from the transfusion recipient who exhibits only mild and transitory signs and symptoms, to more serious cases, with shock, disseminated intravascular coagulation, renal failure, and death. The most severe reactions result from transfusion of incompatible RBCs from donor to recipient leading to rapid intravascular complement-mediated hemolysis. As there are no specific interventions to directly mitigate the effects of acute hemolytic transfusion reaction, only safeguard measures to reduce the risk of an incompatible transfusion are currently employed. Although current safeguards make ABO incompatible transfusions rare in the developed world, AIHTR remains a very high-risk threat for patient populations receiving frequent transfusions. Specifically, individuals with sickle cell disease and severe thalassemias requiring frequent transfusions become “difficult to cross match” due to the accumulation of antibodies against minor antigenic determinants on erythrocytes. In situations of life-threatening anemia (i.e., inadequate oxygen carrying capacity) these patients are not infrequently transfused with the “least incompatible” erythrocytes, because some degree of agglutination occurs with the cross match of all available units. These transfusions are extremely high risk for causing AIHTR, but must be done because the alternative is death by shock. An ideal therapeutic intervention for these individuals would be prophylactic treatment prior to transfusion with “least incompatible” erythrocytes, to decrease the risk of AIHTR. The ability to prevent AIHTR for these patients remains an important unmet medical need. As we have described in this review, the classical pathway of complement plays a central role in AIHTR. Thus, therapeutic inhibition of this system presents a viable strategy to mitigate and prevent AIHTR. Below, we discuss various complement inhibitors, both marketed and in development, and their potential for transfusion medicine and other blood disorders.

### Marketed complement inhibitors

#### C1-inhibitor

C1 esterase inhibitor (C1-INH) is a serine protease inhibitor that was initially demonstrated to regulate the C1 complex by binding and releasing the C1s–C1r–C1r–C1s tetramer from C1q after activation has occurred (40, 41). C1-INH has a broad specificity and also regulates the MBL–MASP2 complex of the lectin pathway of complement as well as serine proteases of the fibrinolytic, coagulation, and kinin systems. C1-INH has been successfully marketed as a replacement therapy for hereditary angioedema (HAE) in which the afflicted individual makes inadequate or defective C1-INH protein. While it is appreciated that HAE is primarily a kinin disease (42), C1-INH has been demonstrated to inhibit complement-mediated disease in several animal models and is currently in clinical trials for trauma and kidney transplantation [reviewed in Ref. (43)]. Recently, a study reported high dose C1-INH concentrate could prevent complement-mediated lysis of RBC in an elderly patient with autoimmune hemolytic anemia (43). While application of C1-INH has potential in complement-mediated disease, aside from HAE, super-physiological amounts of C1-INH are required to see a clinical effect when C1-INH is normally expressed in humans. Infusing high concentrations of C1-INH is of potential concern given C1-INH’s promiscuous inhibition of other serine proteases (43); however, such high doses may be tolerable in an acute intervention setting. While recombinant C1-INH is now available, the purified version of human C1-INH is a blood product requiring extensive purification from large numbers of donors, resulting in an extremely expensive medication that still carries a residual risk of transmitting blood-borne pathogens. The results of the two clinical trials to determine the efficacy of C1-INH are eagerly awaited.

#### Nafamostat

Nafamostat is a synthetic serine protease inhibitor of C1s and other proteases currently marketed for pancreatitis. As is the case with C1-INH, nafamostat inhibits serine proteases other than C1s; thus, it is unclear if its clinical effects can be attributed to suppression of the complement system (44) as opposed to its known anticoagulant properties (45). Currently, nafamostat is only approved in a limited market (Japan and Korea). Other small molecule inhibitors of C1s have also been characterized (46); however, to our knowledge, the role of these inhibitors in transfusion-related indications has not been reported.

#### Eculizumab

Eculizumab is a highly specific, humanized monoclonal antibody that inhibits activation of C5 by the C5 convertase preventing C5a release and MAC formation. Eculizumab has been marketed for the orphan disease paroxysmal nocturnal hemoglobinuria (PNH) preventing the intravascular lysis of RBC that lacks the membrane bound complement regulators CD55 and CD59 (47). Eculizumab is also marketed for atypical hemolytic uremic syndrome (aHUS) (48) and is currently in clinical trials for other indications (44). Eculizumab has also been reported to reverse antibody-mediated complement activation in a case of ABO incompatible kidney and pancreas transplantation (49), and kidney-alone transplantation (50) as well as other off-label uses. Eculizumab is very effective for individuals for PNH; however, it is currently one of the most expensive drugs on the market as a result of its high cost of production and its orphan status. Additionally, since it blocks complement activation at the level of C5, patients utilizing this drug are at increased risk of invasive meningococcal infections and therefore require vaccination. Another drawback for antibody-mediated complement disease indications is that eculizumab acts downstream of C3 and therefore cannot prevent classical pathway generated C3 activation products such as C3a formation or C3b/iC3b opsonization of host cells (e.g., CR1/CR3-mediated clearance of C3b/iC3b-coated RBC in the liver and spleen). This may limit eculizumab’s potential for antibody-mediated, transfusion medicine related indications.

### Complement inhibitors currently in development

A number of complement inhibitors are currently in various stages of pre-clinical and clinical development [please see Ref. (44) for a thorough review]. As one would expect, the compounds currently under pharmaceutical development target a variety of molecules of the complement system including effector molecules and receptor proteins (C5aR, C3, C5, etc.). Given the ubiquitous role of complement in a variety of diseases, these inhibitors have a wide number of indications from transplantation to cancer. Most of these compounds are of the following classes: protein, antibody, or small molecule. Interestingly, there are currently only two peptide compounds in pharmaceutical development. One of the peptides that shows great promise is compstatin, a cyclical peptide that binds C3 preventing C3 activation of all three complement pathways (51). Analogs of compstatin are in various stages of pre-clinical and clinical development for indications such as age-related macular degeneration (AMD), PNH, aHUS, dense deposit disease (DDD), transplantation, and hemodialysis (44). Recently, compstatin analogs have been demonstrated to inhibit hemolysis of PNH RBCs in an *in vitro* system (52), indicating that compstatin may be useful in this RBC disorder. Additionally, compstatin has a significant theoretical advantage over eculizumab by virtue of its ability to block C3 cleavage and thus preventing C3b/iC3b opsonization of RBC leading to extravascular sequestration and hemolysis. While pharmacological blockade of C3 by compstatin positions this molecule as a potent inhibitor of complement, complete shutdown of this critical arm of innate immunity may lead to an increased risk of infection, whether this is a potential issue will become evident as clinical testing of compstatin progresses.

Recently, a mouse monoclonal antibody that targets C1s, known as TNT003, has been demonstrated to prevent complement deposition and RBC destruction via phagocytosis in an *in vitro* assay of CAD (53). TNT003 is currently under clinical development by True North Therapeutics, Inc., as a first-in-class compound designed to specifically inhibit the classical complement pathway. Dosing of TNT009, the humanized monoclonal analog of TNT003, in cynomolgus monkeys demonstrated that a single i.v. injection at 30 mg/kg inhibited the *in vivo* generation of C4a, as determined by ELISA. *Ex vivo* analysis of serum taken from animals receiving this single dose inhibited the hemolysis of IgM-coated sheep RBCs and the deposition of C3b on these cells, as assayed by FACS (54). TNT009 is expected to undergo phase 1 clinical trials in 2015 with an umbrella trial in Europe to test the efficacy of TNT009 in multiple complement disorders (54). The efficacy of TNT009 in clinical trials will be exciting to watch unfold.

### Peptide inhibitors of complement C1

**Figure 5:**
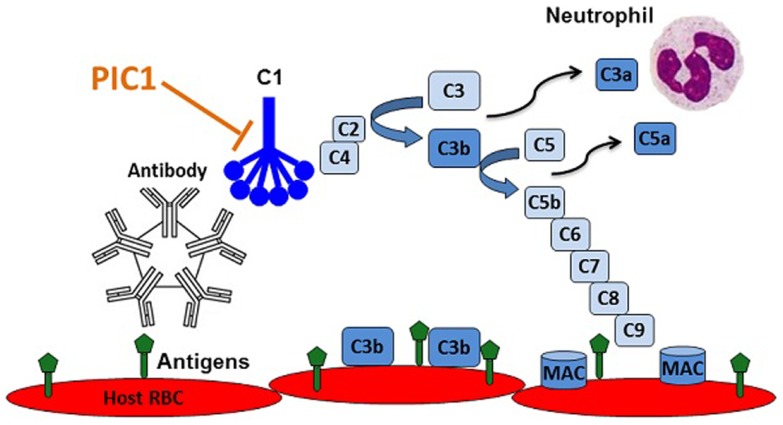
**Recognition of RBC antigens on recipient cells by host derived natural antibodies initiating classical complement via C1 leading to opsonization of host cells with C3b, anaphylatoxin C5a recruitment and activation of neutrophils, and cell lysis by membrane attack complex (MAC) pores**. PIC1 blocks C1 activation at the first step of the amplification cascade.

The fact that several complement inhibitors are currently in pre-clinical and clinical development is a testament to the central role that complement plays in a variety of inflammatory disorders. While the classical pathway of complement instigates antibody-mediated destruction of human RBCs in AIHTR, there is currently no therapeutic treatment for this condition. As outlined in this article, we propose that PIC1 might be an ideal molecule to prevent and treat AIHTR for the following reasons: (i) PIC1 inhibits at the level of the first complement component of classical pathway, C1. Inhibiting classical pathway activation at this step would prevent complement activation at initiation, thus, preventing amplification and downstream complement effector molecule generation (C3a, C5a, MAC, etc.) (Figure 5). (ii) PIC1 binds to the CLR of C1q and MBL. This is opposed to C1-INH and other small molecule serine protease inhibitors, which are known to non-specifically inhibit serine proteases outside of the complement system. Given that PIC1 possesses a differential mechanism of interaction with C1q that does not involve binding to catalytic sites of serine proteases, a higher specificity is expected. (iii) PIC1 does not inhibit the critical immune surveillance functions of the alternative and terminal complement pathway. While inhibitors such as compstatin can potently inhibit C3, effectively shutting down all three complement pathways, this has the potential to predispose individuals to an increased risk of infection. This has been documented for eculizumab that inhibits C5a formation and thus terminal pathway activation increasing the risk of infection by neisserial pathogens. (iv) PIC1 is unique in nature. This is opposed to C1-INH and soluble versions of known complement regulators that are produced endogenously in humans. Thus, molecules such as C1-INH and sCR1 may theoretically require very high doses to achieve a clinical effect. This remains to be determined, however, especially as concerns sCR1, which by virtue of its presence in solution may have additional effects compared to the endogenous membrane bound form. C1-INH and sCR1 appear efficacious in pre-clinical animal models; their efficacy in human trials is currently under evaluation. (v) PIC1 is cheap and easy to produce by virtue of it being a synthetic small peptide. This is in contrast to C1-INH and eculizumab, which are large molecules requiring extensive purification and high production costs. Both these molecules are currently in the top five most expensive drugs in the marketplace for their orphan disease indications, HAE (C1-INH), and eculizumab (PNH and aHUS) (55).

A potential concern for therapeutic inhibition of the classical complement pathway with molecules such as PIC1 or TNT003 is that long-term, systemic inhibition of C1 in humans could pose a risk for developing systemic lupus erythematosus (SLE). Humans lacking functional C1q have been demonstrated to acquire SLE due to the critical role of the classical pathway in maintaining homeostasis through the clearance of cellular debris and immune complexes (7). In the context of an acute disease target such as AIHTR, classical pathway inhibition would not be expected to be a major concern for development of SLE. However, systemic, long-term use of classical pathway inhibitors for chronic disease processes would need to be carefully scrutinized in this regard.

Further development of PIC1 is necessary to test this compound in animal models of classical pathway mediated disease such as AIHTR. To this end, a high potency, water soluble PIC1 derivative has been identified and dose ranging studies in rats are currently underway. Important parameters such as pharmacodynamics, pharmacokinetics, and toxicity of this derivative will be established in these studies. Next, proof-of-concept experiments in the rat model of AIHTR will be performed. Extended dosing and toxicity studies in rats and in a second species (e.g., cynomolgus monkeys) will then need to be completed. This will set the stage for submission of an investigational new drug (IND) application to the FDA under an orphan drug designation. This approach has been successfully utilized for the complement inhibitors eculizumab and C1-INH.

In the US, death from acute hemolytic transfusion reactions has decreased significantly due to numerous safety checks; however, accidental transfusion of incompatible blood between donor and recipient continues to occur due to failure of screening tests or human error (56). Ensuring safe and compatible blood products can be particularly challenging in certain instances such as during emergencies, in a mass casualty setting, or when the supply of blood is limited. Moreover, patients who require frequent blood transfusions may develop multiple antibodies against minor determinants on RBC surfaces decreasing the likelihood that compatible blood can be rapidly identified (56). This is particularly true for patients with sickle cell disease and other individuals suffering from severe chronic anemia requiring many transfusions over their lifetime leading to their development of a wide range of alloantibodies against numerous minor and major erythrocyte determinants (15, 16, 57). The development of these alloantibodies can progress to the point where compatible blood may not be identified in a timely manner, requiring the use of a “least incompatible” RBC unit. Different therapies have been adopted in attempts to minimize the risk from these “least incompatible” units; for example, a “mini-transfusion” challenge can be utilized to try to identify those units, which may lead to a life-threatening complement-mediated acute intravascular hemolytic event before the entire unit is transfused (18). Red cell antigen-sensitized patients, such as those with sickle cell disease or thalassemia, constitute a tremendous challenge for clinicians and the transfusion service particularly in the emergent setting (58). Given the acute hemolysis and hemoglobinemia already occurring for sickle cell disease patients suffering a vasoocclusive crisis, the high risk for worsening their clinical condition with additional AIHTR hemolysis is especially worrisome. In this type of situation, pre-treatment with PIC1 would minimize the risk of AIHTR for sickle cell disease patients meeting a major unmet medical need in an under-served population.

## Conflict of Interest Statement

Drs. Neel K. Krishna and Kenji M. Cunnion have one issued patent and one patent pending on technology reported in the article. The other co-authors declare that the research was conducted in the absence of any commercial or financial relationships that could be construed as a potential conflict of interest.

## References

[1] Cooper NR The classical complement pathway: activation and regulation of the first complement component. Adv Immunol (1985) 37:151–216 10.1016/S0065-2776(08)60340-5 3890478

[2] Kishore U Ghai R Greenhough TJ Shrive AK Bonifati DM Gadjeva MG Structural and functional anatomy of the globular domain of complement protein C1q. Immunol Lett (2004) 85:113–28 10.1016/j.imlet.2004.06.015 15388251 PMC3818097

[3] Rogers J Schultz J Brachova L Lue LF Webster S Bradt B Complement activation by beta-amyloid in Alzheimer disease. Proc Natl Acad Sci U S A (1992) 89:10016–20 10.1073/pnas.89.21.10016 1438191 PMC50268

[4] Tacnet-Delorme P Chevallier S Arlaud GJ Beta-amyloid fibrils activate the C1 complex of complement under physiological conditions: evidence for a binding site for Ab on the C1q globular regions. J Immunol (2001) 167:6374–81 10.4049/jimmunol.167.11.6374 11714802

[5] Mabbott NA Bruce ME Botto M Walport MJ Pepys MB Temporary depletion of complement component C3 or genetic deficiency of C1q significantly delays onset of scrapie. Nat Med (2001) 7:485–7 10.1038/86562 11283677

[6] Klein MA Kaeser PS Schwarz P Weyd H Xenarios I Zinkernagel RM Complement facilitates early prion pathogenesis. Nat Med (2001) 7:410–1 10.1038/86469 11283678

[7] Botto M Walport MJ C1q, autoimmunity and apoptosis. Immunobiology (2002) 205:395–406 10.1078/0171-2985-00141 12396002

[8] Ricklin D Lambris JD Complement-targeted therapeutics. Nat Biotechnol (2007) 25:1265–75 10.1038/nbt1342 17989689 PMC2966895

[9] Jaffe CJ Atkinson JP Frank MM The role of complement in the clearance of cold agglutinin-sensitized erythrocytes in man. J Clin Invest (1976) 58:942–9 10.1172/JCI108547 965497 PMC333257

[10] Wasowska BA Lee CY Halushka MK Baldwin WM New concepts of complement in allorecognition and graft rejection. Cell Immunol (2007) 248:18–30 10.1016/j.cellimm.2007.04.009 17950717 PMC2139895

[11] Ricklin D Lambris JD Complement in immune and inflammatory disorders: pathophysiological mechanisms. J Immunol (2013) 190:3831–8 10.4049/jimmunol.1203200 23564577 PMC3623009

[12] Strobel E Hemolytic transfusion reactions. Transfus Med Hemother (2008) 35:346–53 10.1159/000154811 21512623 PMC3076326

[13] Sève P Philippe P Dufour JF Broussolle C Michel M Autoimmune hemolytic anemia: classification and therapeutic approaches. Expert Rev Hematol (2008) 1:189–204 10.1586/17474086.1.2.189 21082924

[14] Murray NA Roberts IA Haemolytic disease of the newborn. Arch Dis Child Fetal Neonatal Ed (2007) 92:F83–8 10.1136/adc.2005.076794 17337672 PMC2675453

[15] Stowell SR Winkler AM Maier CL Arthur CM Smith NH Girard-Pierce KR Initiation and regulation of complement during hemolytic transfusion reactions. Clin Dev Immunol (2012) 2012:307093 10.1155/2012/307093 23118779 PMC3479954

[16] Engelfriet CP The immune destruction of red cells. Transfus Med (1992) 2:1–6 10.1111/j.1365-3148.1992.tb00128.x 1308459

[17] Davenport RD Hemolytic transfusion reactions. In: Simon TL Snyder EL Solheim BG Stowell CP Strauss RG Petrides M, editors. Rossi’s Principles of Transfusion Medicine. Philadelphia: Lippincott Williams & Wilkins (2009). p. 811–25

[18] Win N Sinha S Lee E Mills W Treatment with intravenous immunoglobulin and steroids may correct severe anemia in hyperhemolytic transfusion reactions: case report and literature review. Transfus Med Rev (2010) 24:64–7 10.1016/j.tmrv.2009.09.006 19962576

[19] Shastri S Doane AM Gonzales J Upadhyayula U Bass DM Prevalence of astroviruses in a children’s hospital. J Clin Microbiol (1998) 36:2571–4 9705394 10.1128/jcm.36.9.2571-2574.1998 PMC105164

[20] Koci MD Moser LA Kelley LA Larsen D Brown CC Schultz-Cherry S Astrovirus induces diarrhea in the absence of inflammation and cell death. J Virol (2003) 77:11798–808 10.1128/JVI.77.21.11798-11808.2003 14557664 PMC229260

[21] Bonaparte RS Hair PS Banthia D Marshall DM Cunnion KM Krishna NK Human astrovirus coat protein inhibits serum complement activation via C1, the first component of the classical pathway. J Virol (2008) 82:817–27 10.1128/JVI.01847-07 17959658 PMC2224607

[22] Matsui SM Greenberg HB Astroviruses. In: Knipe DM Howley PM, editors. Fields Virology. Philadelphia: Lippincott, Williams & Wilkins (2001). p. 875–93

[23] Hair PS Gronemus JQ Crawford KB Salvi VP Cunnion KM Thielens NM Human astrovirus coat protein binds C1q and MBL and inhibits the classical and lectin pathways of complement activation. Mol Immunol (2010) 47:792–8 10.1016/j.molimm.2009.10.006 19896716

[24] van den Berg RH Faber-Krol MC van Wetering S Hiemstra PS Daha MR Inhibition of activation of the classical pathway of complement by human neutrophil defensins. Blood (1998) 92:3898–903 9808583

[25] Groeneveld TW Ramwadhdoebé TH Trouw LA van den Ham DL van der Borden V Drijfhout JW Human neutrophil peptide-1 inhibits both the classical and the lectin pathway of complement activation. Mol Immunol (2007) 44:3608–14 10.1016/j.molimm.2007.03.003 17448537

[26] Gronemus JQ Hair PS Crawford KB Nyalwidhe JO Cunnion KM Krishna NK Potent inhibition of the classical pathway of complement by a novel C1q-binding peptide derived from the human astrovirus coat protein. Mol Immunol (2010) 48:305–13 10.1016/j.molimm.2010.07.012 20728940

[27] Mauriello CT Pallera HK Sharp JA Woltmann JL Jr Qian S Hair PS A novel peptide inhibitor of classical and lectin complement activation including ABO incompatibility. Mol Immunol (2013) 53:132–9 10.1016/j.molimm.2012.07.012 22906481 PMC3630508

[28] Phillips AE Toth J Dodds AW Girija UV Furze CM Pala E Analogous interactions in initiating complexes of the classical and lectin pathways of complement. J Immunol (2009) 182:7708–17 10.4049/jimmunol.0900666 19494295 PMC2824238

[29] Bally I Ancelet S Moriscot C Gonnet F Mantovani A Daniel R Expression of recombinant human complement C1q allows identification of the C1r/C1s-binding sites. Proc Natl Acad Sci U S A (2013) 110:8650–5 10.1073/pnas.1304894110 23650384 PMC3666734

[30] Yazdanbakhsh K Controlling the complement system for prevention of red cell destruction. Curr Opin Hematol (2005) 12:117–22 10.1097/01.moh.0000151712.53957.01 15725901

[31] Yazdanbakhsh K Development of complement therapeutics for inhibition of immune-mediated red cell destruction. Transfusion (2005) 45:122S–9S 10.1111/j.1537-2995.2005.00526.x 16086799 PMC4797633

[32] Yazdanbakhsh K Kang S Tamasauskas D Sung D Scaradavou A Complement receptor 1 inhibitors for prevent ion of immune-mediated red cell destruction: potential use in transfusion therapy. Blood (2003) 101:5046–52 10.1182/blood-2002-10-3068 12576307

[33] Mqadmi A Abdullah Y Yazdanbakhsh K Characterization of complement receptor 1 domains for prevention of complement -mediated red cell destruction. Transfusion (2004) 45:234–44 10.1111/j.1537-2995.2004.04163.x 15660833

[34] Yu J Heck S Debnath A Yazdanbakhsh K Identification of a complement receptor 1 peptide for inhibition of immune hemolysis. Biochem Biophys Res Commun (2007) 353:363–8 10.1016/j.bbrc.2006.12.020 17178110 PMC4804343

[35] Shah TA Mauriello CT Hair PS Sharp JA Kumar PS Lattanzio FA Complement inhibition significantly decreases red blood cell lysis in a rat model of acute intravascular hemolysis. Transfusion (2014). 10.1111/trf.12695 24806146

[36] Tanaka S Suzuki T Nishioka K Assay of classical and alternative pathway activities of murine complement using antibody-sensitized rabbit erythrocytes. J Immunol Methods (1986) 86:161–70 10.1016/0022-1759(86)90448-5 3944473

[37] Hod EA Arinsburg SA Francis RO Hendrickson JE Zimring JC Spitalnik SL Use of mouse models to study the mechanisms and consequences of RBC clearance. Vox Sang (2010) 99:99–111 10.1111/j.1423-0410.2010.01327.x 20345515 PMC3580149

[38] Aptekman PM Bogden AE Characterization of the natural hemagglutinins in normal rat serum associated with a negative phase following tumor implantation. Cancer Res (1956) 16:216–21 13304864

[39] Refaai MA Blumberg N The transfusion dilemma – weighing the known and newly proposed risks of blood transfusions against the uncertain benefits. Best Pract Res Clin Anaesthesiol (2013) 27:17–35 10.1016/j.bpa.2012.12.006 23590913

[40] Sim RB Arlaud GJ Colomb MG C1 inhibitor-dependent dissociation of human complement component C1 bound to immune complexes. Biochem J (1979) 179:449–57 475762 10.1042/bj1790449a PMC1186649

[41] Ziccardi RJ Cooper NR Active disassembly of the first complement component, C-1, by C-1 inactivator. J Immunol (1979) 123:788–92 110880

[42] Zeerleder S C1-inhibitor: more than a serine protease inhibitor. Semin Thromb Hemost (2011) 37:362–74 10.1055/s-0031-1276585 21805442

[43] Wouters D Stephan F Strengers P de Haas M Brouwer C Hagenbeek A C1-esterase inhibitor concentrate rescues erythrocytes from complement-mediated destruction in autoimmune hemolytic anemia. Blood (2013) 121:1242–4 10.1182/blood-2012-11-467209 23411737

[44] Ricklin D Lambris JD Complement in immune and inflammatory disorders: therapeutic interventions. J Immunol (2013) 190:3838–47 10.4049/jimmunol.1203200 PMC3623010 23564578

[45] Maruyama Y Yoshida H Uchino S Yokoyama K Yamamoto H Takinami M Nafamostat mesilate as an anticoagulant during continuous veno-venous hemodialysis: a three-year retrospective cohort study. Int J Artif Organs (2011) 34:571–6 10.5301/IJAO.2011.8535 21786254

[46] Travins JM Ali F Huang H Ballentine SK Khalil E Hufnagel HR Biphenylsulfonyl-thiophene-carboxamidine inhibitors of the complement component C1s. Bioorg Med Chem Lett (2008) 18:1603–6 10.1016/j.bmcl.2008.01.064 18242991

[47] Hillmen P Young NS Schubert J Brodsky RA Socié G Muus P The complement inhibitor eculizumab in paroxysmal nocturnal hemoglobinuria. N Engl J Med (2006) 355:1233–43 10.1056/NEJMoa061648 16990386

[48] Keating GM Eculizumab: a review of its use in atypical haemolytic uraemic syndrome. Drugs (2013) 73:2053–66 10.1007/s40265-013-0147-7 24249647

[49] Biglarnia AR Nilsson B Nilsson T von Zur-Mühlen B Wagner M Berne C Prompt reversal of a severe complement activation by eculizumab in a patient undergoing intentional ABO-incompatible pancreas and kidney transplantation. Transpl Int (2011) 24:e61–6 10.1111/j.1432-2277 21696455

[50] Stewart ZA Collins TE Schlueter AJ Raife TI Holanda DG Nair R Case report: eculizumab rescue of severe accelerated antibody-mediated rejection after ABO-incompatible kidney transplant. Transplant Proc (2012) 44:3033–6 10.1016/j.transproceed.2012.03.053 23195021

[51] Ricklin D Lambris JD Compstatin: a complement inhibitor on its way to clinical application. Adv Exp Med Biol (2008) 632:273–92 19025129 10.1007/978-0-387-78952-1_20 PMC2700864

[52] Risitano AM Ricklin D Huang Y Reis ES Chen H Ricci P Peptide inhibitors of C3 activation as a novel strategy of complement inhibition for the treatment of paroxysmal nocturnal hemoglobinuria. Blood (2014) 27(123):2094–101 10.1182/blood-2013-11-536573 24497537 PMC3968392

[53] Shi J Rose EL Singh A Hussain S Stagliano NE Parry GC TNT003, an inhibitor of the serine protease C1s, prevents complement activation induced by cold agglutinins. Blood (2014) 123:4015–22 10.1182/blood-2014-02-556027 24695853

[54] Stagliano NE Shi J Rose EL Hussain S Singh A Parry GC TNT009, a first-in-class anti-C1s antibody, prevents classical pathway activation in autoantibody-mediated disease. 7th International Conference on Complement Therapeutics Oral Presentation. Olympia (2014).

[55] Herper M The World’s Most Expensive Drugs: Forbes (2010). Available from: http://www.forbes.com/2010/02/19/expensive-drugs-cost-business-healthcare-rare-diseases.html

[56] Janatpour KA Kalmin ND Jensen HM Holland PV Clinical outcomes of ABO-incompatible RBC transfusions. Am J Clin Pathol (2008) 129:276–81 10.1309/VXY1ULAFUY6E6JT3 18208808

[57] Win N Doughty H Telfer P Wild BJ Pearson TC Hyperhemolytic transfusion reaction in sickle cell disease. Transfusion (2001) 41:323–8 10.1046/j.1537-2995.2001.41030323.x 11274584

[58] Nouraie M Lee JS Zhang Y Kanias T Zhao X Xiong Z The relationship between the severity of hemolysis, clinical manifestations and risk of death in 415 patients with sickle cell anemia in the US and Europe. Haematologica (2013) 98:464–72 10.3324/haematol.2012.068965 22983573 PMC3659937

